# How Much Tumor Volume Is Responsible for Development of Clinical Symptoms in Patients With Convexity, Parasagittal, and Falx Meningiomas?

**DOI:** 10.3389/fneur.2021.769656

**Published:** 2021-11-17

**Authors:** Shuhei Yamada, Noriyuki Kijima, Tomoyoshi Nakagawa, Ryuichi Hirayama, Manabu Kinoshita, Naoki Kagawa, Haruhiko Kishima

**Affiliations:** Department of Neurosurgery, Osaka University Graduate School of Medicine, Suita, Japan

**Keywords:** convexity meningioma, falx meningioma, parasagittal meningioma, symptomatic progression, tumor volume

## Abstract

**Purpose:** Meningiomas are the most common primary intracranial neoplasms and clinical symptom appearance depends on their volume and location. This study aimed to identify factors that influence clinical symptoms and to determine a specific threshold tumor volume for the prediction of symptomatic progression in patients with convexity, parasagittal, and falx meningiomas.

**Materials and Methods:** We retrospectively studied patients with radiologically suspected convexity, parasagittal, or falx meningiomas at our institution.

**Results:** The data of three hundred thirty-three patients were analyzed. We further divided patients into two groups based on clinical symptoms: an asymptomatic group (250 cases) and a symptomatic group (83 cases). Univariate analysis revealed significant differences between the groups in terms of sex (*p* = 0.002), age at the time of volumetric analysis (*p* < 0.001), hyperintense lesions on T2-weighted images (*p* = 0.029), peritumoral edema (*p* < 0.001), maximum tumor diameter (*p* < 0.001), and tumor volume (*p* < 0.001). Further multivariate analysis revealed significant differences between the groups in terms of age at the time of volumetric analysis (*p* = 0.002), peritumoral edema (*p* < 0.001), and tumor volume (*p* < 0.001). The receiver operating characteristic curve revealed a threshold tumor volume of 21.1 ml for predicting whether a patient would develop symptoms (sensitivity 0.843, specificity 0.880, an area under the curve 0.919 [95% confidence interval: 0.887–0.951]).

**Conclusion:** We identified factors predictive of clinical symptoms in patients with convexity, parasagittal, and falx meningiomas and determined the first-ever threshold tumor volume for predicting symptomatic progression in such patients.

## Introduction

Meningiomas are the most common primary intracranial tumors, accounting for ~25–38% of all such lesions (1, 2). The number of incidentally discovered meningiomas has increased with the widespread use of neuroimaging modalities such as computed tomography and magnetic resonance imaging (MRI) (3). In fact, radiological studies have revealed that neuroimaging could incidentally reveal suspected meningioma lesions with an incidence ranging from 0.9 to 2.5% in individuals aged in their middle years and older (4, 5). On the other hand, meningiomas are often discovered because of a variety of symptoms, including motor and sensory deficits, cognitive decline, and epilepsy (6–8). However, the factors that determine whether a lesion is symptomatic remain unclear.

Meningiomas are benign neoplasms that can exhibit a variety of growth patterns (9, 10) and, eventually, 67–75% of them enlarge (7, 10, 11). In one study in which the median tumor volume was 35.7 ml (range 1.1–133.1 ml) and 90% (52 patients) were symptomatic, tumor volume was statistically significantly related to the appearance of clinical symptoms (6). In recent meta-analyses, 4.7–8.1% of patients with incidentally discovered intracranial meningiomas developed related symptoms at follow-up visits (11, 12). However, the specific locations of the tumors were not examined in either report. The location of such a tumor is important as it is related to the symptoms a patient will experience (8), as well as the clinical and biological behavior of the tumor (6, 13, 14).

Convexity, parasagittal, and falx meningiomas account for almost 50% of all meningiomas (15). In the report in which the association between tumor volume and clinical symptoms was observed in intracranial meningiomas, nearly half of the cases were skull-base meningiomas (6). Convexity, parasagittal, and falx meningiomas differ from skull-base meningiomas in that they are located in the supratentorial space and do not involve cranial nerves. Thus, the tumor volume that causes clinical symptoms differs between supratentorial and skull-base meningiomas, and it is important to analyze the tumor volume that causes clinical symptoms exclusively for convexity, parasagittal, and falx meningiomas.

The purpose of this retrospective study was to identify factors that influence clinical symptoms and to determine a specific threshold tumor volume for the prediction of symptomatic progression in patients with convexity, parasagittal, and falx meningiomas.

## Materials and Methods

### Study Design and Patient Selection

We conducted a retrospective case-control study of patients with primary radiologically suspected convexity, parasagittal, or falx meningiomas. We collected data from patients whose first visit was from 1990 to 2020 at our institution. We excluded patients diagnosed with neurofibromatosis, those for whom MRI Digital Imaging and Communications in Medicine (DICOM) data were not available, and those whose symptoms were unknown due to insufficient medical records of their first visit. When patients had more than one convexity, parasagittal, or falx meningioma, we selected the largest one for analysis. The Osaka University Clinical Research Review Committee approved the study (approval number 14231) and waived the need for written informed consent, as all data were retrospective.

### Definition of Symptoms

We determined from their medical records whether patients had clinical findings, which we defined as clinical symptoms. When patients exhibited more than one symptom, we selected the one that mainly interfered with their daily life. We further defined neurological symptoms as excluding epilepsy or non-specific symptoms such as headache.

### Volumetric Analysis

We measured the volume of each lesion using the latest MRI DICOM data for asymptomatic patients or the MRI DICOM data at the time of symptom onset for symptomatic patients. We used Horos for macOS to perform the measurements (Horos is a free and open-source code software program that is distributed free of charge under the LGPL license at Horosproject.org and sponsored by Nimble Co LLC d/b/a Purview in Annapolis, MD USA). Using T2-weighed images (T2WIs) or contrast-enhanced T1 weighted images (T1WIs) of ~5 mm slice thickness, we measured the tumor area in each slice by manually tracing the tumor boundary. Thereafter, we multiplied the sum of all the areas by the thickness between slices, including the gaps.

### Tumor Diameter

We used the same MRI DICOM data as for volumetric analysis to measure tumor diameter. The maximum tumor diameter was determined using either axial, coronal, or sagittal images.

### Tumor Location, Side, and Area

The lead author (SY) carefully determined the locations of the lesions via MRI, which was independently confirmed by the senior author (NKi). We divided tumor location in three ways: convexity, parasagittal angle, and falx cerebri; right, and left; frontal, middle, and occipital area. We used “frontal area” for the anterior one-third, “occipital area” for the posterior one-third, and “middle area” for the rest.

### Interpretation of T2-Weighted Images

We classified lesions according to the radiologic characteristics on T2WIs. They were classified as either “T2-hyperintense” or “other” according to the brightness of the lesion. Lesions that were too heterogenous to classify were assigned to the “other” group. One case with a maximum diameter of only 1 mm was excluded from analysis because the lesion was too small to evaluate.

### Statistical Analysis

All statistical analyses were performed using R 3.6.3 for Windows (www.R-project.org; R Foundation for Statistical Computing, Vienna, Austria). Statistical differences for categorical variables were examined using a two-tailed Fisher's exact test. Continuous variables were assessed using the Mann-Whitney U test or the Kruskal-Wallis test adjusted by Bonferroni correction. Multivariate logistic regression analysis was performed with variables that were significant in those univariate analyses. The thresholds were calculated by receiver operating characteristic (ROC) analysis using the distance from the upper left-hand corner (16). Probability values <0.05 were considered significant.

## Results

### Overall Patient Cohort

**Figure 1:**
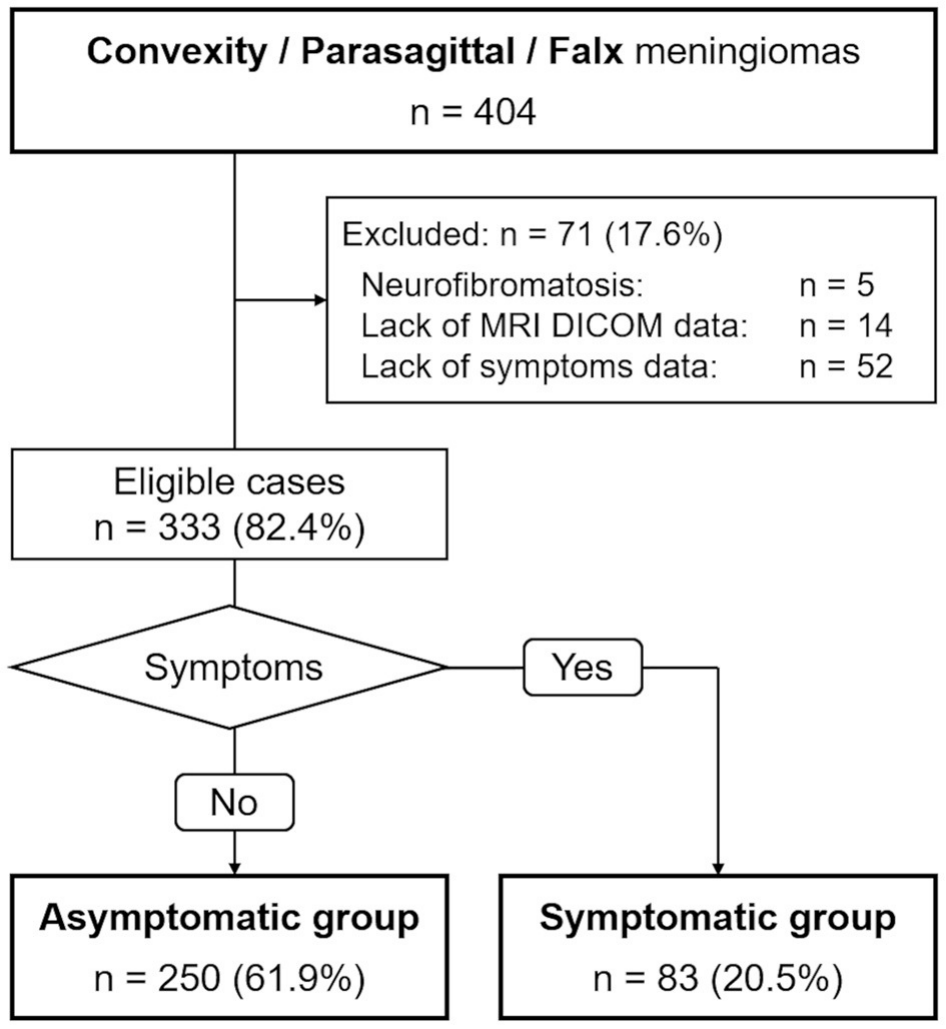
Flow of patient selection and classification.

Figure 1 illustrates the flow of patient selection. The data of 333 patients (84 male and 249 female) were analyzed. The median age at volumetric analysis was 70 years (range 23–90 years). The median tumor volume and the median maximum tumor diameter were 8.2 ml (range 0.1–188.9 ml) and 30 mm (range 5–100 mm), respectively. We further divided patients into two groups based on clinical symptoms: an asymptomatic group (250 cases) and a symptomatic group (83 cases).

### Comparison Between the Asymptomatic and Symptomatic Groups

**Figure 2:**
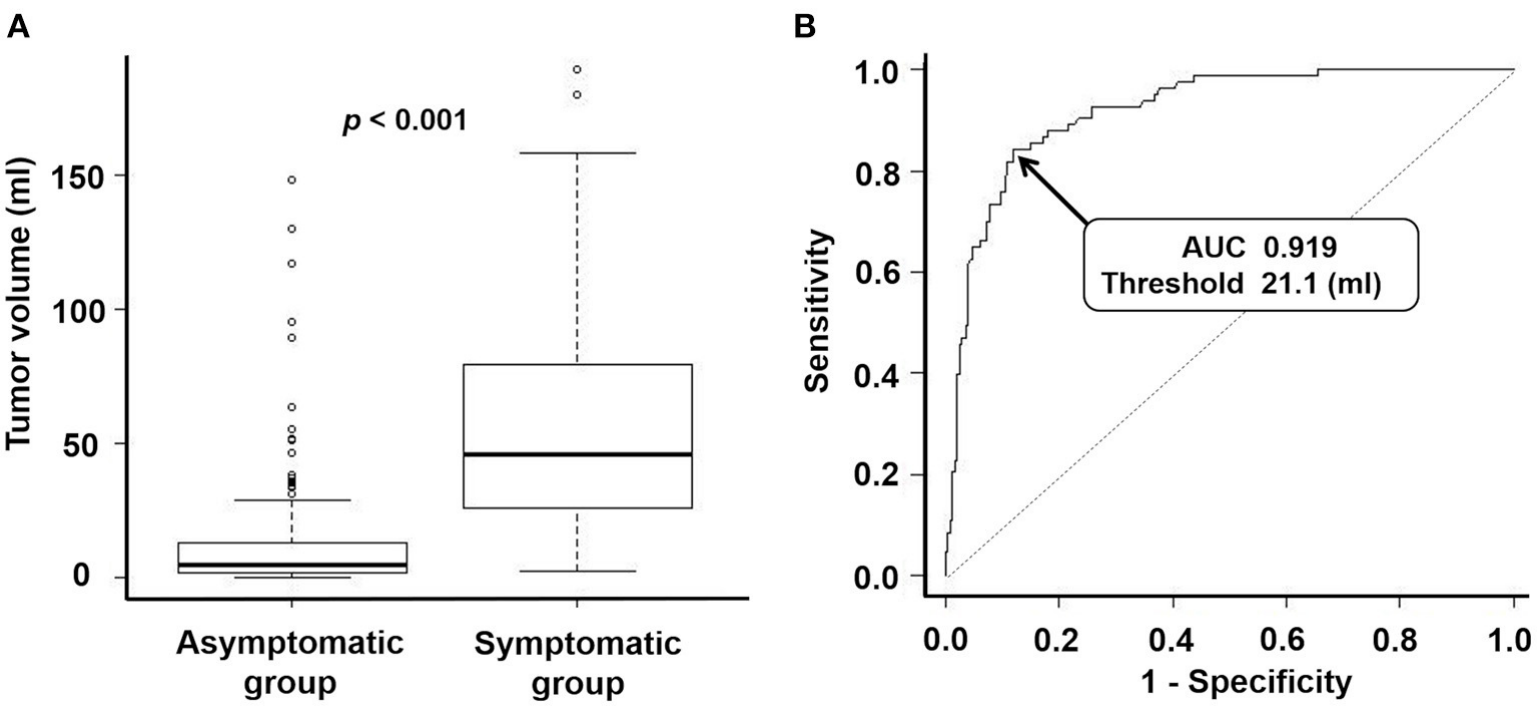
**(A)** Box-and-whisker plots representing tumor volume of an asymptomatic group and symptomatic group. The median tumor volume of the asymptomatic group and the symptomatic group were 5.0 ml and 45.7 ml, respectively. *P*-value for the Mann-Whitney U test: <0.001. **(B)** Receiver operating characteristic curve for predicting which patients will become symptomatic via tumor volume. Area under the curve: 0.919 (95% confidence interval: 0.887–0.951). Threshold for tumor volume: 21.1 ml. Sensitivity: 0.843. Specificity: 0.880.

Table 1 summarizes the characteristics of each group. Univariate analysis revealed significant differences between the groups in terms of sex (*p* = 0.002), age at the time of volumetric analysis (*p* < 0.001), hyperintense lesions on T2WIs (*p* = 0.029), peritumoral edema (*p* < 0.001), maximum tumor diameter (*p* < 0.001), and tumor volume (*p* < 0.001, Figure 2A). For multivariate analysis, the maximum tumor diameter was excluded as it is a similar metric to tumor volume (17). Multivariate analysis revealed significant differences between the groups in terms of age at the time of volumetric analysis (*p* = 0.002), peritumoral edema (*p* < 0.001), and tumor volume (*p* < 0.001). The odds ratio (OR) for peritumoral edema was 5.94 (95% confidence interval [CI]: 2.74–12.86).

**Table 1:** Characteristics of 333 patients and tumors.

**Variable**	**Asymptomatic group** **(*n* = 250)**	**Symptomatic group** **(*n* = 83)**	***p*** **-value**		**OR (95% CI)**
			**Univariate**	**Multivariate**	
Sex (Male/Female)	52/198	32/51	0.002	0.140	1.79 (0.83–3.86)
Age at volumetric analysis (yrs)^*^	72 (23–90)	63 (31–89)	<0.001	0.002	–
Tumor location (Convexity/Parasagittal/Falx)	138/58/54	35/25/23	0.115	–	–
Tumor side (Right/Left)	124/126	41/42	1	–	–
Tumor area (Frontal/Middle/Occipital)	80/125/45	20/49/14	0.324	–	–
MRI T2WI (Hyper/Others)^†^	162/87	65/18	0.029	0.154	1.82 (0.80–4.16)
Multiple lesions (Yes/No)	22/228	4/79	0.345	–	–
Peritumoral edema (Yes/No)	60/190	69/14	<0.001	<0.001	5.94 (2.74–12.86)
Maximum tumor diameter (mm)^*^	24 (5–78)	52 (18–100)	<0.001	–	–
Tumor volume (ml)^*^	5.0 (0.1–148.0)	45.7 (2.8–188.9)	<0.001	<0.001	–

*CI, confidence interval*.

* *Median (range)*.

† *Too small to evaluate: n = 1*.

### Thresholds for Predicting Development of Clinical Symptoms

Patients in the symptomatic group experienced motor deficits (37 cases), epilepsy (18 cases), gait disorder (seven cases), visual impairment (seven cases), cognitive decline (six cases), aphasia (four cases), headaches (two cases), sensory deficits (one case), and a subcutaneous mass (one case). The ROC curve revealed a threshold tumor volume of 21.1 ml for predicting whether a patient would develop symptoms, with a sensitivity of 0.843, a specificity of 0.880, and an area under the curve (AUC) of 0.919 (Figure 2B, 95% CI: 0.887–0.951). In addition, a threshold maximum tumor diameter of 40 mm may also be a reliable marker for predicting which patients will become symptomatic, with a sensitivity of 0.819, a specificity of 0.840, and an AUC of 0.893 (95% CI: 0.856–0.930). For 13 patients in the symptomatic group, we obtained MRI DICOM data when they had been asymptomatic at their first visit. Of these, the 21.1-ml and 40-mm threshold correctly predicted the development of symptoms in seven cases (54%).

### Threshold for Predicting Development of Neurological Symptoms

When focusing only on neurological symptoms (62 cases), the threshold for tumor volume was also 21.1 ml (sensitivity 0.871, specificity 0.880, AUC 0.937 [95% CI: 0.910–0.965]). The threshold for maximum tumor diameter for prediction of neurological symptoms was almost the same as that for all symptoms: 41 mm (sensitivity 0.839, specificity 0.856, AUC 0.914 [95% CI: 0.880–0.948]).

### Comparison by Age at the Time of Volumetric Analysis

When divided into three age groups; <65, 65–74, 75 ≤, the rates of symptomatic patients were 40.7, 24.6, and 9.9%, and the ORs for presenting clinical symptoms were 1 (Reference), 0.47 (95% CI: 0.27–0.84), and 0.16 (95% CI: 0.08–0.33), respectively. In all age groups, the tumor volume was significantly larger in the symptomatic than in the asymptomatic group (*p* < 0.001, Table 2). The threshold for predicting patients in which symptoms would develop was similar: 21.9 ml for patients <65 years, 19.0 ml for patients 64–74 years, and 21.1 ml for patients ≥75 years.

**Table 2:** Comparison by age at the time of volumetric analysis.

**Age at volumetry (yrs)**	Tumor volume (ml)^*^		***p*-value**	**AUC (95% CI)**	**Threshold (ml)**
	**Asymptomatic group** **(*n* = 250)**	**Symptomatic group** **(*n* = 83)**			
<65 (*n* = 108)	7.8 (0.3–148.0)	52.8 (2.8–180.0)	<0.001	0.875 (0.806–0.944)	21.9
65–74 (*n* = 114)	4.2 (0.2–117.1)	45.2 (7.9–188.9)	<0.001	0.921 (0.872–0.970)	19.0
75 ≤ (*n* = 111)	4.3 (0.1–63.6)	40.6 (21.1–103.3)	<0.001	0.971 (0.942–0.999)	21.1

* *Median (range)*.

*AUC, Area under the curve; CI, confidence interval*.

### Comparison by Tumor Location

Tumor volume of the asymptomatic and symptomatic group based on tumor location is shown in Table 3. For all locations, tumor volume was significantly larger in the symptomatic than in the asymptomatic group (*p* < 0.001). Falx meningiomas had a slightly lower threshold for symptomatic progression than other locations.

**Table 3:** Comparison by tumor location.

**Tumor location**	Tumor volume (ml)^*^		***p*-value**	**AUC (95% CI)**	**Threshold (ml)**
	**Asymptomatic group** **(*n* = 250)**	**Symptomatic group** **(*n* = 83)**			
Convexity (*n* = 173)	4.5 (0.1–117.1)	61.2 (2.8–151.6)	<0.001	0.941 (0.897–0.984)	20.5
Parasagittal angle (*n* = 83)	7.0 (0.5–148.0)	29.4 (6.0–180.0)	<0.001	0.868 (0.787–0.950)	18.9
Falx cerebri (*n* = 77)	4.7 (0.3–129.7)	45.7 (7.9–188.9)	<0.001	0.931 (0.876–0.986)	14.0

* *Median (range)*.

*AUC, Area under the curve; CI, confidence interval*.

### Correlation Between Tumor Volume and Tumor Side/Area

Table 4 displays the difference in tumor volume between the asymptomatic and symptomatic groups, depending on the side or area of the tumor. For all areas, the symptomatic group had significantly larger tumor volume than the asymptomatic group (*p* < 0.001). The threshold for predicting patients to develop clinical symptoms was also around 21.1 ml for all areas except the occipital area of the left hemisphere.

**Table 4:** Correlation between tumor side/area and tumor volume (ml).

**Tumor side**	**Right hemisphere** ***n*** **= 165**					**Left hemisphere** ***n*** **= 168**					**Total** ***n*** **= 333**				
**Tumor area**	**Tumor volume** (ml)^*^		***p*-value**	**AUC**	**Threshold (ml)**	**Tumor volume** (ml)^*^		***p*-value**	**AUC**	**Threshold (ml)**	**Tumor volume** (ml)^*^		***p*-value**	**AUC**	**Threshold (ml)**
	**Asymptomatic group** ***n* = 124**	**Symptomatic group** ***n* = 41**				**Asymptomatic group** ***n* = 126**	**Symptomatic group** ***n* = 42**				**Asymptomatic group** ***n* = 250**	**Symptomatic group** ***n* = 83**			
Frontal *n* = 100	4.3 (0.2–148)	82.9 (8.7–131.1)	0.002	0.901 (0.797–1)	17.6	5.0 (0.2–129.7)	53.5 (13.4–114.8)	<0.001	0.918 (0.846–0.990)	22.7	4.8 (0.2–148)	53.5 (8.7–131.1)	<0.001	0.907 (0.847–0.966)	17.6
Middle *n* = 174	4.9 (0.1–89.6)	47.9 (6.0–188.9)	<0.001	0.947 (0.900–0.993)	21.9	6.7 (0.3–37.0)	49.2 (2.8–151.6)	<0.001	0.898 (0.815–0.981)	23.4	5.6 (0.1–89.6)	47.9 (2.8–188.9)	<0.001	0.930 (0.887–0.973)	21.9
Occipital *n* = 59	4.1 (0.7–63.6)	55.0 (7.9–180.0)	<0.001	0.940 (0.848–1)	21.1	3.6 (0.6–35.3)	22.7 (7.7–158.1)	<0.001	0.917 (0.815–1)	12.6	3.9 (0.6–63.6)	36.0 (7.7–180.0)	<0.001	0.925 (0.858–0.993)	12.6
Total *n* = 333	4.6 (0.1–148.0)	47.9 (6.0–188.9)	<0.001	0.928 (0.887–0.968)	21.1	5.5 (0.2–129.7)	45.2 (2.8–158.1)	<0.001	0.907 (0.857–0.957)	22.7	5.0 (0.1–148.0)	45.7 (2.8–188.9)	<0.001	0.919 (0.887–0.951)	21.1
*p*-value	0.969	0.859	–	–	–	0.489	0.504	–	–	–	0.775	0.976	–	–	–

* *Median (range)*.

*AUC, Area under the curve; CI, confidence interval*.

### Comparison by WHO Grade

Thirty-three patients in the asymptomatic and 77 in the symptomatic group received surgical treatment. Of the 110 patients, the meningiomas of 90 (81.8%) were World Health Organization (WHO) grade I, 10 (9.1%) were WHO grade II, 3 (2.7%) were WHO grade III, and 7 (6.4%) were not mentioned in WHO grade. There were no significant differences between patients with WHO grade I and those with WHO grade II/III meningiomas, except in the maximum tumor diameter, which was larger in the latter than in the former (Table 5).

**Table 5:** Comparison of WHO grade I and grade II/III meningioma.

**Variable**	**WHO grade**		***p*-value**
	**I (*n* = 90)**	**II/III (*n* = 13)**	
Sex (Male/Female)	35/55	5/8	1
Age at volumetry (yrs)^*^	63 (23–83)	67 (41–77)	0.471
Tumor location (Convexity/Parasagittal/Falx)	42/19/29	7/5/1	0.113
Tumor side (Right/Left)	41/49	7/6	0.767
Tumor area (Frontal/Middle/Occipital)	24/50/16	2/8/3	0.778
MRI T2WI (Hyper/Others)	75/15	8/5	0.125
Multiple lesions (Yes/No)	8/82	0/13	0.592
Peritumoral edema (Yes/No)	65/25	12/1	0.176
Maximum tumor diameter (mm)^*^	48 (18–82)	63 (22–100)	0.044
Tumor volume (ml)^*^	37.6 (2.8–188.9)	71.2 (5.0–123.9)	0.111

* *Median (range)*.

*WHO, World Health Organization*.

## Discussion

In the present study, we identified factors that are related to clinical symptoms of patients with convexity, parasagittal, and falx meningiomas, and, to our knowledge, we determined the first-ever threshold of tumor volume for predicting symptomatic progression in such patients. This may allow clinicians to predict when a growing, asymptomatic meningioma will become symptomatic. Currently, observation is the first choice for management of patients with asymptomatic meningiomas (18); these results may be useful to determine the necessity of treatment and its appropriate timing.

In this study, we also determined a threshold maximum tumor diameter of 40 mm for the prediction of symptomatic progression of patients (sensitivity 0.819, specificity 0.840, AUC 0.893). However, a tumor volume of 21.1 ml was a more accurate threshold (sensitivity 0.843, specificity 0.880, AUC 0.919). Maximum tumor diameter and tumor volume are highly correlated (17). However, tumors do not always grow in the direction of their maximum diameter, which may be why we discovered tumor volume to be the more accurate predictive factor. Maximum tumor diameter is one of the most convenient clinical metrics; however, our results indicate that the development and widespread use of a simple method to measure tumor volume is needed.

Meningiomas manifest in a variety of symptoms (8), including non-specific symptoms such as headaches and dizziness (7). Such non-specific symptoms make clinicians wonder if interventions such as surgery or radiotherapy are required for the existing meningioma. In this study, we also calculated thresholds for tumor volume and maximum tumor diameter to predict the development of neurological symptoms. However, the thresholds were similar to those for all clinical symptoms. Therefore, it may be less important for clinicians to examine whether non-specific symptoms are caused by a given meningioma.

The hemisphere in which glioblastomas and strokes occur affects the symptoms that the patient experiences (19, 20); however, in this study of meningiomas, we detected no differences between hemispheres. This may be because few patients presented with cognitive decline (7%) or aphasia (5%). The low threshold of the tumor volume only in the occipital area of the left hemisphere may be due to the small number of symptomatic patients itself. Falx meningiomas also had a lower threshold for symptomatic progression than other locations, but as in previous studies (21–23), the number of cases may not have been sufficient. Therefore, further large-scale studies are needed to validate our location-specific findings.

In multiple meta-analyses (11, 24), a T2-hyperintense sign was correlated with radiological progression, and peritumoral edema was the only imaging metric that correlated with symptomatic progression, which was confirmed in this study. Since T2WIs may be appropriate for follow-up of untreated meningiomas (25), symptom-related radiological indicators that do not require contrast-enhanced T1WIs are needed.

We should note that this study has several limitations. The first is the fact that this was a single-center, retrospective study conducted in Japan. As Japan has the largest number of MRIs per unit population in the world (26), a larger proportion of small, asymptomatic meningiomas may be detected than in other countries. This would have lowered the thresholds of tumor volume and maximum tumor diameter for predicting symptomatic progression of patients in this study. The second limitation is the possibility of errors in volumetric measurements. Volumetric measurement may be inaccurate especially for small tumors (27), and manual segmentation may be inconsistent (28). Finally, this study was conducted on radiologically presumed meningiomas; therefore, 2.9–3.4% of our study population may actually have had other tumors (10, 29).

## Conclusion

In the present study, we identified factors predictive of clinical symptoms in patients with convexity, parasagittal, and falx meningiomas and, to our knowledge, determined the first-ever threshold tumor volume for predicting symptomatic progression in such patients. These results may be useful in allowing clinicians to estimate when a growing, asymptomatic meningioma will develop clinical symptoms, thereby improving management of patients with the disease.

## Data Availability Statement

The original contributions presented in the study are included in the article/supplementary material, further inquiries can be directed to the corresponding author.

## Ethics Statement

The studies involving human participants were reviewed and approved by Osaka University Clinical Research Review Committee. Written informed consent for participation was not required for this study in accordance with the national legislation and the institutional requirements.

## Author Contributions

SY and NKi contributed to the concept, drafting, and writing the manuscript. TN contributed to the acquisition of data. RH and NKa contributed to the manuscript editing. MK contributed to the provision of insightful thought and design. HK contributed to the study supervision. All authors contributed to the article and approved the submitted version.

## Conflict of Interest

The authors declare that the research was conducted in the absence of any commercial or financial relationships that could be construed as a potential conflict of interest.

## Publisher's Note

All claims expressed in this article are solely those of the authors and do not necessarily represent those of their affiliated organizations, or those of the publisher, the editors and the reviewers. Any product that may be evaluated in this article, or claim that may be made by its manufacturer, is not guaranteed or endorsed by the publisher.

## Abbreviations

ROC: receiver operating characteristic
OR: odds ratio
CI: confidence interval
AUC: area under the curve
WHO: World Health Organization.
